# Painful swollen leg – think beyond deep vein thrombosis or Baker's cyst

**DOI:** 10.1186/1477-7819-6-6

**Published:** 2008-01-18

**Authors:** Buchi RB Arumilli, Vinayagam Lenin Babu, Ashok S Paul

**Affiliations:** 1 The Regional Sarcoma Centre, Manchester Royal Infirmary, Oxford Road, Manchester, M13 9WL, UK

## Abstract

**Background:**

The diagnosis of deep vein thrombosis of leg is very common in clinical practice. Not infrequently a range of pathologies are diagnosed after excluding a thrombosis, often after a period of anticoagulation.

**Case presentation:**

This is a report of three patients who presented with a painful swollen leg and were initially treated as a deep vein thrombosis or a baker's cyst, but later diagnosed as a pleomorphic sarcoma, a malignant giant cell tumor of the muscle and a myxoid liposarcoma. A brief review of such similar reports and the relevant literature is presented.

**Conclusion:**

A painful swollen leg is a common clinical scenario and though rare, tumors must be thought of without any delay, in a duplex negative, low risk deep vein thrombosis situation.

## Background

Painful swollen leg is a common clinical scenario. Deep vein thrombosis (DVT) often presents as a painful swollen leg and prompt management is vital to prevent fatal pulmonary embolism. The common differential diagnoses include cellulitis and a ruptured baker's cyst [1]. Rare pathologies with a similar clinical picture to venous thrombosis of calf [2] and dual pathologies have been reported [1,3] including tumors [4]. Careful evaluation is needed to avoid inappropriate management and vitally a catastrophic delay in initiating appropriate treatment. We report three case histories of patients managed initially as a DVT of calf or a baker's cyst and later referred to our centre with a provisional diagnosis of a soft tissue tumor.

## Case presentation

### Case 1

**Figure 1:**
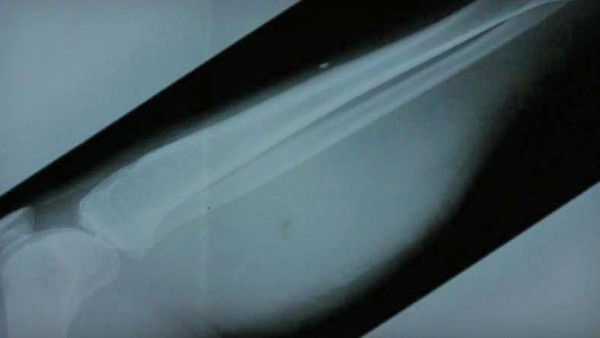
Plain X ray of the leg (Case 1) showing the massive soft tissue swelling of calf.

**Figure 2:**
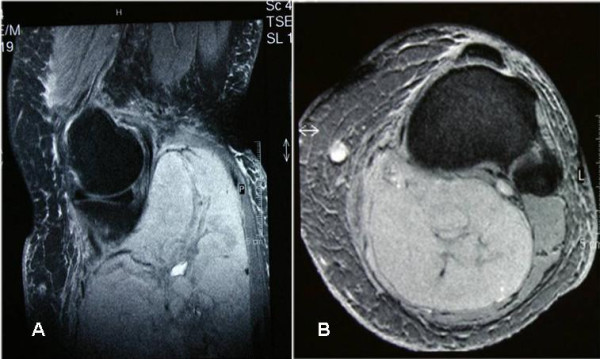
A & B – MR images (longitudinal & transverse sections) of the left leg (case 1) showing a massive pleomorphic sarcoma involving the whole posterior compartment.

A 70 year old female presented to general practitioner with complaints of pain in left knee and calf. Initial knee radiographs showed early osteoarthritis. As there was associated calf tenderness she was admitted for further investigations. All blood parameters were normal. D-dimers at the time of admission were 440 ng/ml. She was categorized as moderate risk for a DVT on clinical examination. Anticoagulation was initiated suspecting a DVT and the Duplex scan of the leg was inconclusive. The pain settled but swelling persisted and the patient was managing her regular activities. After six months since the initial presentation she was referred to us for increasing swelling of the left leg. There was marked swelling with venous congestion (difference of circumference of 8 cm from right calf). After full length X-rays of leg (Figure 1), she had an MR scan of left knee and leg. The scan revealed a soft tissue mass showing marked enhancement, arising from the soleus muscle extending to popliteal fossa and involving the entire posterior compartment of leg (Figure 2A &2B). Ultrasound guided biopsy confirmed a high grade pleomorphic sarcoma. Locally the tumor was encasing the neurovascular bundle at the popliteal fossa. No metastases were discovered. After a total of 11 months from the onset of symptoms she underwent an above knee amputation on left side and is currently disease free with regular follow-up.

### Case 2

A 59 year female was investigated for a possible venous thrombosis of calf at the emergency department after she presented with a painful swollen left proximal calf. She was categorized as a low risk for DVT on clinical examination. D-dimers were 260 ng/ml and Duplex imaging was equivocal. She was started on treatment dose of heparin.

After 3 weeks of anticoagulation there was evidence of a lump in the left popliteal fossa and ultrasound scan of the area revealed a solid soft-tissue mass. She had distal paraesthesia in the foot without any motor weakness. CT scan revealed a soft tissue lump behind knee & proximal calf and she was referred to our centre.

**Figure 3:**
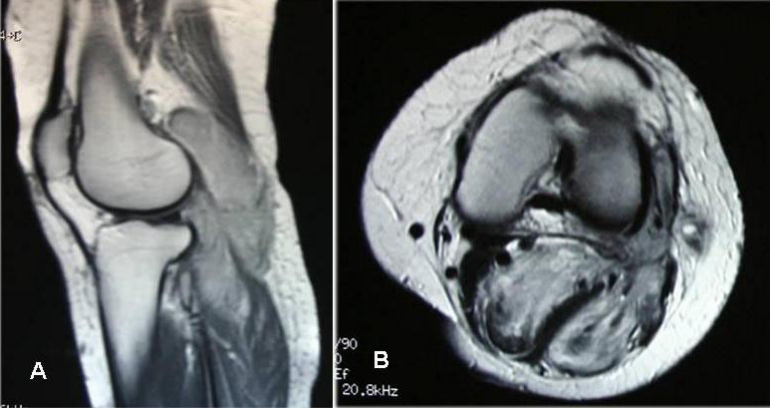
A & B – MR image (longitudinal & transverse sections) of the left knee & leg (Case 2) showing the soft tissue malignant giant cell tumor arising from the muscle posteriorly.

On examination she had a diffuse swelling behind the knee with a good range of painless movement. Ultrasound guided biopsy revealed an extra-articular diffuse malignant giant cell tumor arising from muscle. On MR imaging (Figure 3A &3B) there was evidence of invasion into the knee joint posteriorly. CT thorax and abdomen were normal. An extensive local excision was performed. She is currently disease free and is under regular follow-up.

### Case 3

A 69 year male was seen for a swollen and painful right calf following a minor trauma. This was treated initially with physiotherapy and the pain settled. Two years later he was further investigated for a similar episode, this time to rule out a DVT. The D-dimers were normal and a Doppler scan ruled out a DVT, but a baker's cyst was diagnosed. Following this episode the symptoms never settled and he was later reviewed for a sudden increase in size of the calf 4 years later. There was an 8 × 8 cm diffuse but discrete swelling over the lateral aspect of his calf.

**Figure 4:**
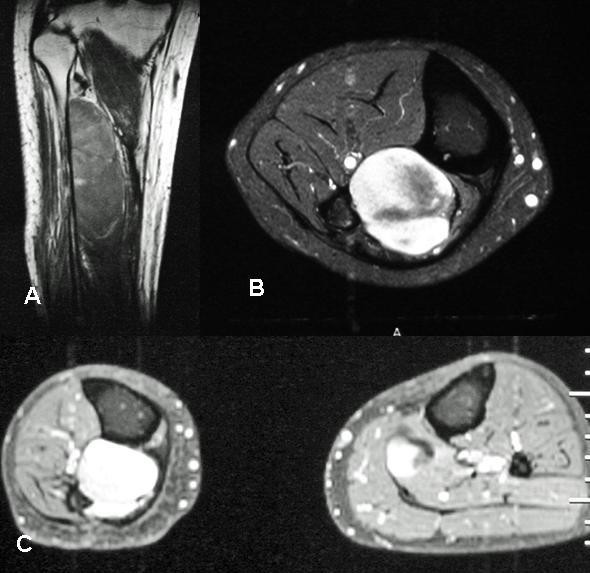
A& B – MR image (longitudinal & transverse) of the right leg showing a large myxoid liposarcoma (Case 3). C) – MR Transverse sections of both legs (Case 3) showing a secondary lesion in the soleus muscle on the left side along with an aggressive recurrence of the primary on the right side.

An MR scan revealed a heterogenous soft tissue mass probably of fatty origin in the posterior compartment measuring 10 × 25 cm (Figure 4A &4B). This was confirmed to be a low grade Myxoid Liposarcoma on biopsy. After a wide local excision patient was clear of disease for 3 years but developed multiple recurrences along with a secondary lesion in the soleus muscle on the opposite leg (Figure 4C). He underwent palliative excision with radiotherapy.

## Discussion

Painful swollen leg is a common clinical scenario for a wide range of pathology. The initial management in the majority is to start anticoagulation and arrange a venous duplex scan, as the priority is to rule out a DVT. But only one third of the first episodes of a venous thrombosis are spontaneous [5]. Patients should be stratified into low, intermediate or high-risk categories before treating for a venous clot [6]. A new evidence based protocol combining clinical probability and D-dimer evaluation has proven effective in deciding when to initiate anticoagulation [7]. D-dimer levels as a stand-alone test for the diagnosis of DVT is not recommended as it can be elevated in many other conditions [8]. When used along with the clinical risk assessment score, the combination had negative predictive values of 97–100% for a DVT [9,10]. Contrast venography is the gold standard for venous disease [11] but is not performed routinely as it is invasive. Duplex scanning of limbs is the common alternative but has disadvantages of being highly operator dependent [11] and poor sensitivity for calf DVTs ranging between 54–93% [12].

In case of the first patient, a moderate risk for DVT clinically along with moderately elevated D-dimers prompted anticoagulation. But after an initial equivocal Duplex, a repeat scan or venography should have been performed to establish or exclude a DVT. There was some relief of pain which made both the patient and physician less concerned. Only after 6 months when the swelling was much worse, an alarm was raised. In the second patient a DVT was unlikely given the low clinical risk and the D-dimer level of 260 ng/ml. Further evaluation should have been done, as an alternate diagnosis was more likely. In her case the lump was much more proximal to be appreciated within 3 weeks of initial presentation. The combination of low pre test probability of a DVT along with an inconclusive Duplex scan in these patients must have prompted further investigations. In the final patient of this series, a Duplex was sensitive enough to diagnose a Baker's cyst but was unable to detect a co-existing solid soft-tissue swelling which was probably small by the time. A general ultrasound in this patient must have a given a better information regarding the underlying pathology.

The discovery of nonvascular disease is not an infrequent finding of duplex scan. Baker's cyst is the commonest non-vascular abnormality found in patients undergoing duplex scan for a suspected DVT (3%) [1]. The other differential diagnoses include cellulitis, hematoma, tumors [2], venous or arterial aneurysms [13] and connective tissue disorders [14]. Tumors are a rare but an important differential diagnosis in such patients. Sixty percent of soft-tissue sarcomas arise in the extremities, 70 % occur in the lower limb and mostly in the thigh. As a rule of thumb any mass over 5 cm in size arising beneath the level of deep fascia should be considered a sarcoma unless proven otherwise [15]. On clinical examination of the calf a major difficulty is when a swelling is deep to the deep fascia making it difficult to appreciate as a lump. In a series of 200 patients investigated for venous disease, eight patients were found to have previously undiagnosed lower extremity masses of which three were malignant [4]. Special investigations (angiography, CT or MR scanning) were necessary to establish diagnosis (sarcomas, lymphoma, aneurysm, hematoma, abscess and cyst) in 31% of patients referred as DVT to one unit over a 2 year period [2]. Another issue complicating diagnosis is a coexisting venous thrombosis secondary to obstruction or stasis from an underlying local cause. Lewis et al reported a patient who had a popliteal vein thrombosis and 4 weeks later was found to have a leiomyosarcoma arising from the popliteal vein [3]. In low risk patients when a duplex scan fails to reveal a thrombus especially in the calf, a general ultrasound would provide useful additional information. It is non-invasive and has an important role in differentiating a cystic and a solid swelling and its size thereby providing relevant information for further management [16].

## Conclusion

The purpose of this case series is to highlight the need to be vigilant before diagnosing a DVT in low risk patients presenting with a painful swollen calf and make clinicians realize that d-dimer levels alone could be misleading as levels could be moderately elevated in other pathologies. A delay in diagnosing tumors could affect the overall prognosis hence further investigations or imaging should be considered without any delay.

## Competing interests

The author(s) declare that they have no competing interests.

## Authors' contributions

**BA **drafted the manuscript and performed the literature review. **VL **has compiled the figures and collected the necessary data of patients. **AP **conceived of the project and coordinated the final draft along with proof reading. All authors read and approved final manuscript.
